# Automatic Detection and Classification of Audio Events for Road Surveillance Applications

**DOI:** 10.3390/s18061858

**Published:** 2018-06-06

**Authors:** Noor Almaadeed, Muhammad Asim, Somaya Al-Maadeed, Ahmed Bouridane, Azeddine Beghdadi

**Affiliations:** 1 Department of Computer Science and Engineering, College of Engineering, Qatar University, Doha 2713, Qatar; muhammad.asim@qu.edu.qa (M.A.); s_alali@qu.edu.qa (S.A.-M.); 2 Department of Computer and Information Sciences, Northumbria University Newcastle, Newcastle upon Tyne NE1 8ST, UK; ahmed.bouridane@northumbria.ac.uk; 3 L2TI, Institut Galilée, Université Paris 13, Sorbonne Paris Cité 99, Avenue J.B. Clément, 93430 Villetaneuse, France; beghdadi@univ-paris13.fr

**Keywords:** event detection, visual surveillance, tire skidding, car crashes, hazardous events

## Abstract

This work investigates the problem of detecting hazardous events on roads by designing an audio surveillance system that automatically detects perilous situations such as car crashes and tire skidding. In recent years, research has shown several visual surveillance systems that have been proposed for road monitoring to detect accidents with an aim to improve safety procedures in emergency cases. However, the visual information alone cannot detect certain events such as car crashes and tire skidding, especially under adverse and visually cluttered weather conditions such as snowfall, rain, and fog. Consequently, the incorporation of microphones and audio event detectors based on audio processing can significantly enhance the detection accuracy of such surveillance systems. This paper proposes to combine time-domain, frequency-domain, and joint time-frequency features extracted from a class of quadratic time-frequency distributions (QTFDs) to detect events on roads through audio analysis and processing. Experiments were carried out using a publicly available dataset. The experimental results conform the effectiveness of the proposed approach for detecting hazardous events on roads as demonstrated by 7% improvement of accuracy rate when compared against methods that use individual temporal and spectral features.

## 1. Introduction

An increase of road accident rates has become a source of increased casualties and deaths leading to a global road safety crisis [1]. A total of 52,160 Road Traffic accidents (RTAs) were recorded during the year 2000 in the state of Qatar. Among them, there were 1130 injuries and 85 fatalities. The data consisted on RTAs was collected from the traffic department, Qatar. Almost 53% of the death victims were in the age 10–40 years old, and the remaining 53% who died due to RTAs were in the age of 10–19 years old [2]. Furthermore, traffic accidents are amongst the major sources of public death in the US [3] for individuals of age 11 and of every age from 16 through 24 in 2014. The number of motor-vehicle deaths reported in 2016 was 40,200, a 6% increase from 2015, and the first time the total annual fatality has exceeded 40,000 since 2007 [4].

The most common reason of death in a road accident is the belated delivery or absence of first aid due to the delay of the notification of the accident reaching the nearest hospital or ambulance team. Every minute of delay to provide emergency medical aid to injured crash victims can significantly decrease their survival rate. For instance, an analysis conducted in [5] showed that a reduction by 1 min in the response time is correlated with a six-percent difference in survival rate. A quick and timely medical response by emergency care services is, therefore, required to reach the accident spot to deliver timely care to the accident victims.

Thus, a preferred approach for reduction in road traffic death rate, is to decrease the unnecessary delays in information to reach the emergency responders [6]. A six-percent fatality reduction would be possible if all time delays of the notification to emergency responders can be eliminated [7]. To address this issue, traditional vehicular sensor systems, for instance OnStar, can detect car accidents by means of accelerometer and airbag control modules and notify appropriate emergency services immediately by means of built-in cellular radio sensors [8]. Automated Crash Notification (ACN) systems exploit the telemetric data from the collided vehicles to inform the emergency services in order to reduce fatalities from car accidents [9]. Visual sensors (surveillance cameras) are also widely used to monitor driver and vehicle behavior by tracking vehicle trajectories near traffic lights or on highways to monitor traffic flow and road disruptions [10,11] A robust visual monitoring system would detect the abnormal events and instantly inform the relevant authority [12].

However, not all vehicles or roads are equipped with automated accident or damage detection sensors. Furthermore, an analysis based solely on visual data is insufficient and prone to errors [13]. Indeed, the performance of CCTV cameras is limited under adverse weather conditions and is highly sensitive to sudden lighting changes, reflections, and shadow [14]. Moreover, the imaging systems have a limited depth-of-field. An activity that occurs within a certain range from the camera remains in focus, while objects closer or away, out of the range appear as blurred or out-of-focus in the image [15]. In such cases, the visual information available from CCTV cameras is not sufficient to reliably track or monitor the activities of vehicles or to accurately detect hazardous situations. Certain abnormal events, such as gunshots, squealing brakes and screaming cannot be accurately detected from visual information only but have very distinguishing acoustic signatures. Such kind of events can be efficiently detected by analyzing audio streams acquired by microphones [16]. Furthermore, it may happen that the abnormal events occur outside the range of a camera (field of view) or occluded by obstacles, making them almost impossible to detect by visual analytics. In such scenarios, audio stream analysis can provide complementary information, which may improve the reliability and robustness of the video surveillance system [17].

Furthermore, CCTV cameras facilitate the collection of information about individuals and thus can lead to increased risk of unintentional browsing or abuse of surveillance data [18]. On the other hand, audio-based systems can be an alternative solution in situations where CCTV cameras are not permitted due to privacy concerns (e.g., public toilets) [19]. Audio monitoring systems can be employed either alone or in combination with existing surveillance infrastructures [20]. In fact, Internet protocol (IP) cameras, which are commonly used for surveillance applications, are typically equipped with embedded microphones, making them multisensory and multimodal fusion systems that are capable of utilizing both audio and visual information.

Standard video cameras are almost useless at night because of the scarce ambient illumination and glare from car headlights. By contrast, audio analysis systems are unaffected under varying lighting conditions. However, this task becomes challenging and the performance may reduce in an open and noisy environment such as a highway. An audio analysis system in such an environment faces a high level of non-stationary background noise in addition to potentially relevant sound events. The sounds of interest to be recognized are interrupted by background noises, making it hard to isolate them from the environmental noise that is often non-stationary. The energy of audio events of interest may be low compared with the unwanted non-stationary noise [21].

This paper proposes an efficient method for audio event detection for traffic surveillance application. The proposed system performs automatic detection and classification of two types of road hazardous situations: car crashes (CC) and tire skidding (TS) in the presence of environmental noise by analyzing the audio streams captured by microphones. The TS event is considered as an abnormal event because a frequent occurrence of this event is a sign of a dangerous and busy road state, which may require attention from traffic personnel to ensure safety. The problem of audio events detection and classification such as gunshots, explosions, and screams has been addressed in several previous studies [15,16,22,23]. Although various audio surveillance system setups and additional audio features have been explored, most of the approaches proposed in the previous studies are based on the conventional methods of modeling short-term power spectrum features such as the Mel-frequency cepstral coefficient (MFCC) [24], using either Gaussian mixture models (GMMs) or Hidden Markov models (HMMs) [20].

The state-of-the-art approaches for audio-based surveillance can be classified into two main categories, depending upon the architecture used for classification [16]. Methods residing in the primary category rely on frame by frame operation. The input signal is divided into small chunks of frames, from which characteristic features (MFCCs or wavelet-based coefficients) are extracted. These features are then processed by a classifier to make decisions. For example, Vacher et al. [25] detected screams or gunshots by employing GMM-based classifiers trained on Wavelet based cepstral coefficients. Similarly, Clavel et al. [26] did the same using 49 different sets of features. Valenzise et al. [27] used this approach to model background sounds. In [28], the automatic detection and recognition of impulsive sounds were proposed utilizing a median filter and linear spectral band features. Six types of impulsive events were classified using both GMM and HMM classifiers, and the results were assessed. The authors in [26] used short time energy, MFCCs, some spectral features and their derivatives and their combination with a GMM classifier to detect abnormal audio events in continuous recordings of public places. Later a more advanced scheme was designed in [29] to detect impulsive sounds such as gunshots and screams. In addition, this system is also able to localize the positions of such sound events in a public place. Small improvements were achieved by adding more features, i.e., temporal, spectral distribution, and correlation-based features. Similarly, a two-stage recognition schema was proposed in [30]. The incoming audio signal is processed to classify it as either a normal sound (metro station environment) or abnormal sound (gunshot, scream or explosion) using MFCC features and an HMM classifier. In case if it is determined to be abnormal, the system can then identify the type of abnormality using maximum-likelihood classification.

In the second category of audio surveillance techniques, more complex architectures have been proposed. For instance, the authors in [31] presented a combination of two different classification techniques: GMM and a support vector machine (SVM) classifier, to detect shout events in a public transport vehicle. A three-stage integrated system based on a GMM-classifier was proposed in [32], in which the first stage categorizes the incoming signal as a volcanic (normal speech or a shout) or nonvolcanic (gunshot, explosion, background noise) event. In the second stage, the sounds are further classified into normal or screamed (speech) and in case if it is nonvolcanic event, classified into non-threating background sound or atypical sound event of interest. Finally, in the third stage, the system identifies the type of hazardous situation the incoming signal belongs to. In [33], an automatic recognizer was proposed that extracts a wide set of features from input signals and classifies them into abnormal events of interest, i.e., screams, gunshot, or broken glass, using two classifiers with rejection options. Their approach consists of combining the decisions of both classifiers to reduce the false detection rate.

The proposed technique employs a short-time analysis based on features observed in the temporal, spectral and the joint time–frequency (*t*, *f*) domain, extracted from quadratic time–frequency distributions (QTFDs), for sound detection and classification. Whereas previous studies have either used a combination of temporal and spectral features [13,17] or (*t*, *f*)-based techniques [22,23] alone for non-stationary signal classification such as audio data, this work is novel in the sense that we have used a combination of *t*-, *f*- and (*t*, *f*)-domain features. In addition, we have extracted (*t*, *f*)-features from high resolution and robust QTFDs, whereas in previous studies time-frequency features are extracted from conventional spectrograms. We have initially evaluated the resolution and classification performances of different QTFDs, allowing us to select the best amongst them, i.e., the Extended modified beta distribution (EMBD). The performance of the proposed approach is evaluated on the publicly available MIVIA dataset [13], which contains two types of hazardous events: car crashes and tire skidding. The main aim of this work is to correctly classify events of these two types in the presence of background noise on the highways. For the performance evaluation, the resulting classification results are compared with some of state-of-the-art approaches [13,19].

## 2. Methods and Materials

This section reviews some basic notions on advanced signal processing tools and the relevant features that are used for audio events classification including temporal, spectral, and (*t*, *f*) features. We also describe the proposed methodology including the MIVIA dataset and the experimental setup.

### 2.1. The Proposed Approach

**Figure 1:**
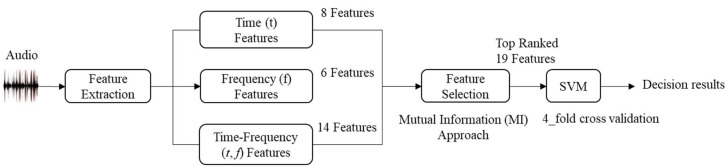
Proposed methodology for detecting acoustic anomalies. The input signal is subdivided into small frames, and features are extracted in the time, frequency, and joint time-frequency domains. The highest ranked features among the computed features are selected.

The audio signals of the MIVIA dataset (to be discussed in Section 2.4) were processed to extract 200 CC segments, 200 TS segments, and the same number of segments containing background sounds. Each segment has a duration of 0.75 s, which is the minimum duration of an audio segment in the available database [34]. The proposed architecture for audio analysis and classification includes four steps: (1) extraction of temporal and spectral features, (2) extraction of joint (*t*, *f*)-domain features, (3) feature selection and (4) classification. Figure 1 illustrates the proposed methodology used in this study in detecting events of interest. A detailed explanation of each layer is given below.

#### 2.1.1. Extraction of Audio Features

In this work, twenty-eight features were analyzed for each audio segment. The features employed can be broadly classified into three categories: (1) *t*-domain features, (2) *f*-domain features, and (3) joint (*t*, *f*)-domain features. These features are listed in Table 1 and Table 3. The *t*-domain features are extracted from the temporal-domain, the *f*-domain features are extracted from the spectral-domain, and the (*t*, *f*)-domain features are extracted from the TFD’s of the audio signals, respectively.

#### 2.1.2. Temporal, Spectral, and Energy Features

The feature extraction stage plays a crucial role in our proposed automatic event classification. Conventionally, the temporal and spectral characteristics of a signal were mainly used for feature extraction, analysis, and processing of the underlying signal. Typical time- and frequency-domain features include mean, variance, skewness, kurtosis, entropy, spectral flux, mean frequency, etc., as depicted in Table 1.

The processing of audio signals is usually performed on a frame-by-frame basis in order to take into consideration real-time application constraints and the non-stationarity nature of the signals. Indeed, for audio processing, the delay to receive signals is critical and should be kept as low as required by end users. Moreover, unlike video streams, audio signals can be more highly non-stationary, exhibiting abrupt variations on a time scale of a few milliseconds.

Thus, to account for its non-stationarity and high variability, an audio stream is subdivided into small and partially overlapped segments of a certain time-period TF [16]. The selection of the size of the TF of the temporal window analysis is critical. Therefore, a tradeoff has to be found when analyzing low and high frequency components of the signal. If the TF is too long, the processing would fail to capture the most rapid variations in the spectral content of the signal. The system would not consider high-frequency components, as they will be normalized over the long time-interval. Meanwhile, an excessively small frame size will not be able to provide sufficient resolution to consider low-frequency signal components. A short TF slot does not provide sufficient information for extracting the relevant features, making the signal analysis more ambiguous and prone to discrimination errors. The more rapid the spectral content of a signal change is, the shorter the frame length must be. Once the signal is segmented into frames, frame-level features are computed in the time and frequency domains. In our experiment, the audio signals were sampled at 32 kHz and divided into segments of 0.75 s in length. The signals were divided into frames using a Hamming window of 200 ms and a 50% overlap. The set of low-level features based on temporal and spectral characteristics previously employed in [35,36] is utilized in this study. More details on these *t*- and *f*-domain features are reported in Table 1.

When considering signals of different types such as CC (an implosive sound with short duration), TS (a long-sustained sound that lasts for several seconds), and BN—it is expected that the signals would have different statistical details such as the probability distribution functions (PDFs). The PDFs can be characterized by their moments such as mean, variance, zero-crossing rate, skewness, kurtosis, coefficient of variation etc., and their relative energies. The frequency-domain features are extracted from the spectrum of audio signals to discriminate the signals based on their spectral variations. In this study, we have utilized the most commonly used spectral features such are spectral flux, spectral flatness, centroid, Roll-Off, spectral entropy, and maximum power of the frequency bands [37]. The spectral flux characterizes the change in the signal’s frequency spectrum with time. Spectrum roll-off identifies the skewness of the signal’s frequency spectrum. The spectrum centroid, as visible from its name, is defined as the center of power spectrum distribution of a signal. It gives different values for the normal (BN) and abnormal events such as (CC). The spectral entropy and flatness measures the degree of randomness of the signal energy distribution.

Individual *t*- and *f*-domain features can be exploited to discriminate among signal classes that have different energy distributions. For instance, the narrow and wideband signals can be efficiently differentiated by the conventional f-domain features such as spectral flatness, However, such features cannot clearly discriminate among signals having similar energy distributions in the f-domain. For example, the spectral flatness cannot efficiently discriminate two wideband signals; i.e., white noise from linear frequency modulated (LFM) signals with completely different (*t*, *f*) signatures. Authors in [38] suggested that these limitations can be overcome by extending the time- and frequency-domain features to the (*t*, *f*) domain.

#### 2.1.3. Time-Frequency Domain based Approach for Event Detection and Classification

**Figure 2:**
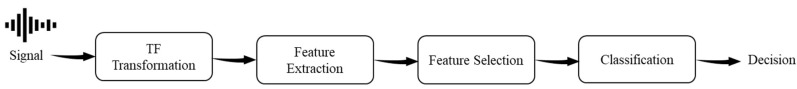
Time-frequency (TF) approach for pattern classification.

Previous papers have shown that the existing solutions using non-stationary signal analysis and processing can be improved by exploiting the (*t*, *f*) attributes of such signals. In addition, (*t*, *f*)-based techniques have been reported to outperform the conventional techniques (time-and frequency-domain) in the detection and classification of non-stationary signals [39,40]. This is because the (*t*, *f*) distributions (TFDs) provide more discriminative and effective features that cannot be extracted from temporal or spectral representations. For instance, the instantaneous frequencies (IFs) and instantaneous amplitudes of the components of the signal. The fore-mentioned effectiveness of the (*t*, *f*)-representations for the analysis, processing and classification of non-stationary signals directed us to a (*t*, *f*)-based pattern recognition approach illustrated in Figure 2. A detailed description of such TF methods is given in [38].

A t-domain representation refers to the variation in the amplitude of signal at each time to determine the signal changes over time in order to confirm the presence of the signal by indicating its start and end times. On the other hand, a spectral representation gives information about the frequency components of the signal, including their magnitudes, the start and end frequencies, and the bandwidth. The *t*-domain representation does not provide information about the frequency components present in the signal. Similarly, the frequency domain representation does not show at which times these components are present. By contrast, the TFD indicates the start and stop times of its components, including their frequency range and variations over time [41]. Thus, TF analysis can effectively illustrate the non-stationary aspects of signal, discontinuities, and repeating patterns, whereas the previous two approaches are not as effective for this purpose.

**Figure 3:**
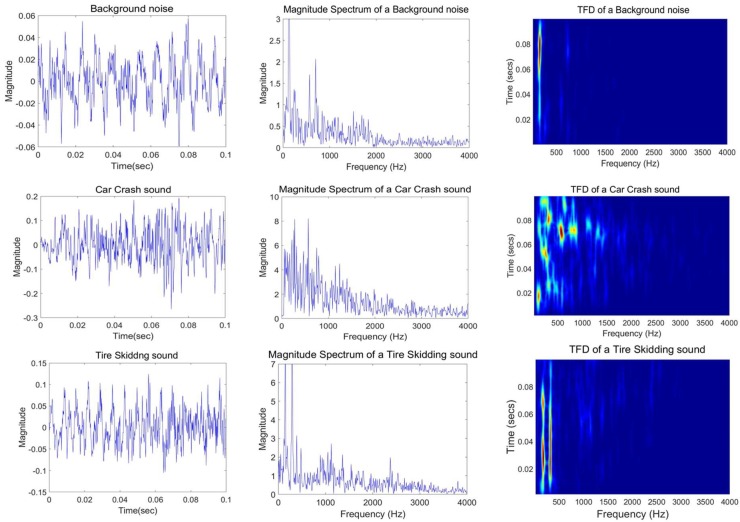
Time, frequency, and TF representations of a Background noise (BN) segment (**1st row**), a Car Crash (CC) sound (**2nd row**), and a Tire Skidding (TS) sound (**3rd row**). The TF representations were generated using the extended modified-B distribution (EMBD) with as σ = 0.9 and β = 0.01, with a lag window length of 355.

Because CC and TS signals are highly variant, they can be best represented by TFDs. A TFD efficiently describes the spread of a signal’s energy over the two-dimensional TF space instead of time or frequency space alone. Figure 3 shows examples of a short segment (0.1 s sampled at 8 kHz) of background road noise (BN) and CC and TS sound events in the t domain (left-hand plots), the f domain (center plots) and the joint (*t*, *f*) domain (right-hand plots) using the (EMBD).

These TFD plots clearly illustrate the differences between the signals by providing extra information about the signal’s frequency components including their temporal variations and instantaneous frequencies (IFs). This representation better clarifies the nature of the signals by highlighting the pattern of frequency changes and thus facilitating the classification of the audio events. This additional informative description of audio signals motivates us to extract relevant (*t*, *f*) features from the TFDs for signal classification purpose. Moreover, an analysis of the spectral plots of several TS and BN segments reveals that they have high similarity in energy distributions in the f-domain. Therefore, the temporal and spectral features do not provide a selective separation of audio signals.

Unlike the previous TF based audio classification approaches where a conventional spectrogram is often used for signal analysis and feature extraction, the TF approach used in this work includes finding an optimal TFD that best represents the signal, followed by feature extraction, and finally assigning these (*t*, *f*) features to the relevant classes.

##### Extraction of Time-Frequency Features

TFD’s contain significant amounts of information and to avoid the dimensionality problem, not all the (*t*, *f*) points are used for feature extraction. Therefore, a small set of features must be extracted from TFDs that best describes information relevant for signal classification. The authors in [40,41] presented a methodology for extending t- and f-domain features to the (*t*, *f*)-domain.

To extract (*t*, *f*)-based features, it is necessary to select an optimal TFD that best represents the signals of interest. The selected TFD must be able to highlight the frequency patterns in the signals that can be exploited to discriminate among different classes. Previous studies have shown that the most common TFDs for this purpose are QTFDs including the Wigner–Ville distribution (WVD), the smoothed WVD (SWVD), the modified-B distribution (MBD), and the spectrogram (SPEC) [38]. In this work, relevant (*t*, *f*) features were extracted from the (*t*, *f*)-representations formed from the QTFDs of audio signals for the classification of audio anomalies into M classes. Each audio segment was first transformed into the (*t*, *f*)-domain using a QTFD denoted by $\rho_{y}$(*t*, *f*) and written as

(1) $$\rho_{y}\left(t,f\right)=\int_{-\infty }^{\infty }\int_{-\infty }^{\infty }\int_{-\infty }^{\infty }e^{j2\pi v\left(u-t\right)}g\left(v,\tau \right)\;y\left(t+\frac{\tau }{2}\right)y^{*}\left(t-\frac{\tau }{2}\right)e^{-j2\pi f\tau }dvdud\tau$$

where y(t) is the analytic signal associated with the real-valued signal x(t), obtained using the Hilbert transform, {H(t)}, y(t)=x(t)+jH {x(t)}, and g(v,τ) represents a 2-dimensional kernel that controls the characteristics of the TFD.

The WVD is a QTFD that can be computed by setting *g*(v,τ)=1 and can be expressed as

(2) $$W_{y}\left(t,f\right)=\int_{\infty }^{\infty }y\left(t+\frac{\tau }{2}\right)y^{*}\left(t-\frac{\tau }{2}\right)e^{-j2\pi f\tau }d\tau$$

whereas the EMBD can be computed using the form for g(v,τ) given below:

(3) $$g\left(v,\tau \right)=\frac{{\left|\Gamma \left(\beta +j\pi v\right)\right|}^{2}}{\Gamma^{2}\left(\beta \right)}\frac{{\left|\Gamma \left(\alpha +j\pi \tau \right)\right|}^{2}}{\Gamma^{2}\left(\alpha \right)}$$

where Γ(·) denoted the gamma function and β is known as a smoothing parameter.

Similarly, different QTFDs can be computed by altering the kernel functions, adopted to particular kind of signals.

The clarity of a TFD representation is a principal factor to ensure that relevant (*t*, *f*) features can be extracted. The presence of cross terms adversely affects the features that are extracted from a TFD image. Therefore, to select a suitable TFD for audio analysis with minimal cross terms and high resolution, various QTFDs were computed and assessed on the audio signals of interest. Among them, the separable kernel TFD i.e., the EMBD, which is the extended version of the MBD, was found to best represent the audio signals, as illustrated in Figure 3. The superior performance of the separable kernel TFD (EMBD) can be explained by its flexibility to exclusively include smoothing over time and frequency axes, thereby resulting in better characterization of signals whose components are varying in time or frequency. Moreover, the EMBD has shown superior performance in analyzing real-life signals [38]. Therefore, separable TFDs are generally better the than the spectrogram. 

Previous studies have shown that (*t*, *f*)-based features proposed in [40,42] yielded excellent classification results for non-stationary signals (EEG signal). In this work, because of the non-stationarity of the signals of interest, we have utilized (*t*, *f*) features in combination with temporal and spectral features. The (*t*, *f*) features were extracted from the QTFDs, whereas the corresponding time-and frequency-domain features are extracted from their time- and frequency-domain representations, respectively. The various temporal and spectral features considered in this study are reported in Table 2, whereas, the time-frequency features are represented in the following.

##### Extension of Temporal and Spectral Features to Joint (*t*, *f*)-Domain Features

For the sake of completeness and understanding of the proposed method, the most relevant (*t*, *f*) features are recalled below.

- The standard Mean of a temporal signal *x*[*n*] can be extended to (*t*, *f*)-domain as:
  (4) $${\text{µ}}_{\left(t,f\right)}=\frac{1}{NL}\sum_{n}\sum_{m}\rho_{y}\left[n,m\right]$$
  where $\rho_{y}$ represents the TFD matrix of size (N×L) associated with the analytic signal *y*. Similarly, the STD, Skewness, Kurtosis measure and the Coefficient of variation are respectively expressed as,
- The Standard variance:
  (5) $$\sigma_{\left(t,f\right)}^{2}=\frac{1}{NL}\sum_{n}\sum_{m}{\left(\rho_{y}\left[n,m\right]-{\text{µ}}_{\left(t,f\right)}\right)}^{2}$$
- The Skewness measure:
  (6) $$\gamma_{\left(t,f\right)}=\frac{1}{NL\sigma_{\left(t,f\right)}^{3}}\sum_{n}\sum_{m}{\left(\rho_{y}\left[n,m\right]-{\text{µ}}_{\left(t,f\right)}\right)}^{3}$$
- The Kurtosis measure as:
  (7) $$K_{\left(t,f\right)}=\frac{1}{NL\sigma_{\left(t,f\right)}^{4}}\sum_{n}\sum_{m}{\left(\rho_{y}\left[n,m\right]-{\text{µ}}_{\left(t,f\right)}\right)}^{4}$$
- The Coefficient of Variation:
  (8) $$C_{\left(t,f\right)}=\frac{\sqrt{\sigma_{\left(t,f\right)}^{2}}}{{µ}_{\left(t,f\right)}}$$

Similarly, the spectral features could be extended to joint time-frequency features, as shown below. In the following we recall the expressions of Spectral Flux, Spectral Flatness, Spectral Entropy, Instantaneous Frequency (IF), Instantaneous Amplitude (IA), TFD complexity measure, TFD concentration measure and geometric features such as Complex Hull and Aspect ratio.

- The Spectral flux in the (*t*, *f*)-domain can be estimated from the TFD matrix $\rho_{y}$ of size (K×L) using the following expression.
  (9) $$SF_{\left(t,f\right)}=\sum_{i=1}^{K}\sum_{j=1}^{L}{\left(\rho_{y}\left[i,j\right]-\rho_{y}\left[i+K,j+L\right]\right)}^{2}$$
- The Spectral Flatness in the (*t*, *f*)-domain is defined as follows.
  (10) $$SFT_{\left(t,f\right)}=KL\;\frac{{\left(\Pi_{i=1}^{K}\Pi_{j=1}^{L}|\rho_{y}\left[i,j\right]|\right)}^{\frac{1}{KL}}}{\sum_{j=1}^{K}\;\sum_{j=1}^{L}|\rho_{y}\left[i,j\right]|}$$
- The Spectral Entropy in the (*t*, *f*)-domain can be expressed as,
  (11) $$SE_{\left(t,f\right)}=-\sum_{i=1}^{K}\sum_{j=1}^{L}\frac{\rho_{y}\left[i,j\right]}{\sum_{i}\;\sum_{j}\;\rho_{y}\left[i,j\right]}log_{2}\left(\frac{\rho_{y}\left[i,j\right]}{\sum_{i}\;\sum_{j}\;\rho_{y}\left[i,j\right]}\right)$$
- The Instantaneous Frequency (IF) of a mono-component signal corresponds to the peak frequency in the (*t*, *f*) plane and given by,
  (12) $$IF\left(t\right)=arg_{f}max\left(\rho_{y}\left[t,f\right]\right)$$
- Similarly, the Instantaneous Amplitude (IA) of a mono-component signal corresponds to the amplitude of the peak frequency in the (*t*, *f*) plane and can be expressed as,
  (13) $$IA\left(t\right)=\rho_{y}\left(t,f\left(t\right)\right)$$
- The (*t*, *f*) complexity measure, a SVD-based feature is derived from the Shannon entropy of the singular values of the TFD matrix $\rho_{y}$. The TFD matrix is first decomposed into two subspaces given as,
  $$\rho_{y}=UIV^{*}$$
  where U and V are orthogonal matrixes of order M×N. Here I is a diagonal matrix and values of I are known as the singular values of the TFD matrix.
  (14) $$CM=-\sum_{n=1}^{K}{Ῑ}_{n}\;log_{2}{Ῑ}_{n}$$
  here ${Ῑ}_{n}$ represents the nth normalized singular value.
- The TFD concentration measure of a multicomponent audio signal can be represented as,
  (15) $$M={\left(\sum_{i=1}^{K}\sum_{j=1}^{L}|\rho_{y}\left[i,j\right]{|}^{1/2}\right)}^{2}$$
- The geometric features such as Convex Hull and Aspect ratio are image descriptors, extracted from a TFD matrix, when considered as an image. These features give the information regarding the geometry of energy concentration regions in the (*t*, *f*) plane. The segmentation technique such as water-shed is first applied on the TFD matrix to detect the regions where most of the TFD energy appears, and then a binary segmented image $p_{y}^{s}$ is generated, where s represents the number of (*t*, *f*) regions. Finally, the geometric features are extracted from the moments of $p_{y}^{s}$, given by,
  (16) $$IM_{nm}=\sum_{i=1}^{K}\sum_{j=1}^{L}i^{n}j^{m}\;\rho_{y}^{s}\left[i,j\right]$$
  where *n* and *m* = 0, 1, 2, …

Finally, the two shape features such as TF convex hull or area and the aspect ratio are extracted from the moments and are defined by Area $=IM_{00}$ and Aspect ratio = $IM_{20}-IM_{02}$.

### 2.2. Proposed Feature Selection Scheme

Feature selection is an essential part of any classification schema. Among the *t*-, *f*- and (*t*, *f*)-domain features computed above, a compact subset of superior features was selected, as listed in Table 3. The relevant features listed in Table 3 are ranked and selected using the mutual information approach defined in [43]. If the mutual information between two variables is large, it means they are closely related. The purpose of this scheme is to select a set of features (m) from a large set K that have maximum relevance and minimum redundancy among themselves [43].

Maximum relevance identifies the features that satisfy (17). It approximates the maximum dependency D(K,s) based on the mean values of all mutual information values between each feature $x_{i}$ and the target class s (e.g., the CC, TS, or BN class):

(17) $$\max\;D\left(K,s\right),\;D=\frac{1}{\left|K\right|}\sum_{x_{i}\in K}I\left(x_{i};s\right)$$

where K represents set of features (which contains m−1 features) and I(x;s) is the mutual information between features x and the target class s and is defined as follows:

(18) $$I\left(K;c\right)=\int \;\int \;P\left(K;s\right)log\frac{P\left(K;s\right)}{P\left(K\right)P\left(s\right)}dKds$$

Minimum redundancy means selecting relevant features with as little redundancy as possible:

(19) $$\min\;R\left(K\right),\;R=\frac{1}{{\left|K\right|}^{2}}\sum_{X_{i},Y_{j}\in K}I\left(X_{i},Y_{j}\right)$$

where $I\left(X_{i};Y_{j}\right)$ is the mutual information between two features $X_{i}$ and $Y_{j}$ and is defined as shown below:

(20) $$I\left(x;y\right)=\int \;\int \;P\left(x;y\right)log\frac{P\left(x;y\right)}{P\left(x\right)P\left(y\right)}dxdy$$

where P(x) and P(y) are the probability density function (PDF) of two random variables x and y.

### 2.3. SVM-Based Classifier

The performance of the proposed set of features is evaluated using the LIBSVM classifier defined in [44]. Because the proposed system is intended to solve a multiclass classification problem, we have used a kernelized version of a multiclass SVM classifier, i.e., with the radial basis function (RBF) kernel, since it was found to yield better results than the linear kernel in our experiments. The parameters of the kernel function were fine-tuned using the grid-search procedure [45], which involves testing different parameter values for the SVM kernel. As far as the choice of the SVM classifier is concerned, it is preferred over the known classifiers like k-nearest neighbors (KNN) and GMM in most of the sound recognition systems [46]. Moreover, it is an easily implemented method, needs fewer parameters to be adjusted, and is found to give superior results using low training data. The output of the SVM classifier characterizes whether the input sound signal *x*[*n*] is a CC or TS event or is a BN segment. 

### 2.4. Data Set and Experiment Setup

In this paper, we have used the publicly available dataset for road surveillance applications described in [13]. The dataset is composed of two hazardous road events: CCs and TSs where the sounds of interest are not isolated. To enable the consideration of such abnormal events occurring under real-world conditions, the signals are superimposed on various background noises including crowd, cars passing nearby, and traffic jams. From the given dataset, we have extracted three sets of events: CC, TS, and BN, respectively. Each class contains 200 segments with each having a length of 0.75 s (the average temporal length of events of interest), without any editing or aligning considerations. The segment duration of 0.75 s was chosen because it is the minimum length corresponding to the shortest events in the database. Furthermore, when longer audio segments are analyzed, the resulting features are more consistent with long-term audio characteristics.

The *t*- and *f*-domain features were extracted by subdividing the audio segments into frames using a Hamming window of 200 ms, corresponding to 1024 PCM samples, and a 50% overlap. The choice of $T_{f}=200\;\mathrm{ms}$ is reasonable because the acoustic signals of interest are characterized by a long duration. Thus, the features should be computed over a longer frame size than the one commonly used in speech processing and recognition (20–30 ms). Two consecutive frames were defined to overlap by 50% of their length to avoid discontinuity and good stitching of the audio streams. The (*t*, *f*) features were extracted from the TFD of each audio segment of length T, whereas the corresponding *t*- and *f*-domain features were extracted from the *t*- and *f*-domain representations, respectively. In this work, the signals were analyzed using five TFDs: the MBD, the EMBD, the short-time Fourier transform (STFT), the SPEC and the SWVD. The simulations for the MBD and EMBD were carried out with parameters chosen as σ = 0.9 and β = 0.01, with a lag window length of 355. Similarly, a Hanning window with a length of 71 samples is utilized for the SWVD, STFT, and SPEC distributions. The simulation results were performed in MATLAB. 

For the experiments, we have split the data as necessary to apply the *N*-fold cross-validation technique. Cross-validation is a useful technique used to assess the performance of a classification system on distinct sets of data under different conditions. In this technique, the dataset of interest is separated into training and testing folds whose constituent samples are independent of each other, meaning that the test data are not seen during training. The data contained in (*N*−1) folds are used to train the classification model, and the remaining fold is then used to test the system. Finally, the results of the *N* different test experiments are averaged. In this study, a multi-class SVM classifier with the RBF kernel was trained using a combination of *t*-, f- and (*t*, *f*)-domain features. The MIVIA road dataset was utilized using the same split with *k* = 4 folds, each containing 200 events per class. Then, each possible set of (*k*−1) folds was used as a training set, and the remaining fold was given as the test set. The results of all k tests were then averaged to obtain the final result.

## 3. Results and Discussions

In order to evaluate the relevance of the set of features considered in the study, the proposed features were ranked in accordance with the criteria defined in Section 2. Table 3 shows all *t*-, *f*- and (*t*, *f*)-domain features as well as the set of features that were selected based on the minimum redundancy and maximum relevance criteria [43]. The total classification accuracy is calculated using the n top-ranked features, where n=1, …, 28. Through experiments, we observed that the best total classification accuracy was achieved using the 19 top-ranked features listed in Table 3.

The performance of the proposed approach is evaluated using several different measures: (1) the recognition rate (RR), i.e., the rate of correctly identified events; (2) the false positive rate (FPR), i.e., rate of falsely identified abnormal events which actually belongs to background sounds; (3) the missed detection rate (MDR), i.e., the rate of undetected abnormal events; and (4) the error rate (ER), i.e., the rate of detection of abnormal events but are falsely classified. Using these measures, the classification matrix achieved using the proposed approach is illustrated in Table 4.

We have compared the results with those achieved using the MFCC features extracted as proposed in [47] and the system proposed in [13]. By considering a combination of *t*-, f- and (*t*, *f*)-domain features, the proposed system achieved an average RR of 95% in the presence of background noise, with an MDR of 2.25%. In the case of the MFCC features extracted as proposed in [47], the system achieved an average RR equal to 88% and an MDR of 2.5%. For comparison, an average RR of 80.25% or 82% with an MDR of 19% or 17.25%, respectively, was achieved using the bag-of-words (BoW) approach proposed in [13] when the system was configured with K=64 clusters (number of words) and was implemented based on MFCC features or a combination of spectral and temporal features, respectively.

A detection system is considered to be very sensitive if it has a low false negative rate (FNR), meaning that when an event occurs, it is almost certain to be detected. On the other hand, a system is considered to be very specific if it has a low FPR, meaning that when an event is reported, it has almost certainly occurred, with few false alarms. The proposed system is extremely sensitive compared with the sets of features proposed in [13], as indicated by the MDR of 2.25%. It also has better specificity than either the Bark or the MFCC features, although its specificity is lower than that of the temporal-spectral feature set proposed in [13], by a small margin.

**Figure 4:**
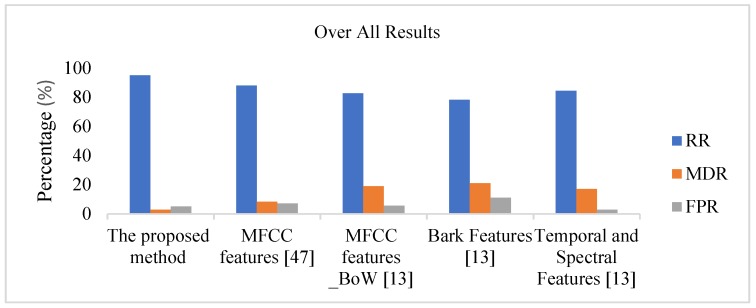
Comparison of performance results.

We also observe that in the proposed approach, the rejection error (undetected events of interest) is less than the interclass error (wrongly classified events). By contrast, for the method proposed in [13], we observe that the major source of classification error is the rejection error, whereas the interclass error is low. In Table 5, we have summarized the achieved results in comparison with the features set that is proposed in [13] for a system configuration with K=1024 clusters. We observe that the average RR and MDR for the proposed set of features are much better than those for both the MFCC features based systems proposed in [47] and [13], as depicted in Figure 4.

The proposed system, with its combination with temporal, spectral, and joint (*t*, *f*) features, shows higher robustness to traffic noise than the Bark and MFCC features proposed in [13,46] but lower robustness than the bag-of-words approach when applied based on spectral and temporal features [13]. However, further studies on the use of advanced QTFDs to extract additional (*t*, *f*) features could improve the robustness to noise.

Furthermore, in order to confirm the improvement in the RR caused by the combination of the three typologies of features, Table 6 illustrates the accuracy achieved by the *t*-domain features, f-domain features, and joint (*t*, *f*) features, in addition to their combination without feature selection and after feature selection. Refer to Table 4, among the 19 top ranked features, 10 features are (*t*, *f*) features, followed by 5 f-domain features, and 4 *t*-domain features. This shows that the (*t*, *f*) features are the most relevant features for audio classification and are supported by the results depicted in Table 6. The selection of the top 19 features significantly reduces the computation cost of our audio classification system.

**Figure 5:**
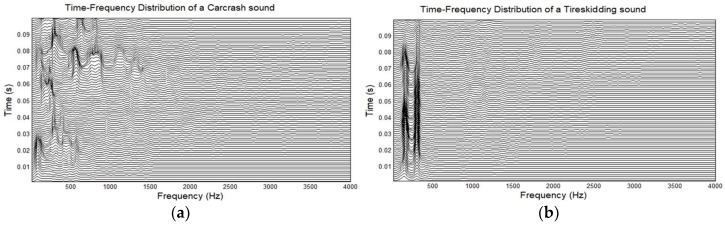
Illustration of higher entropy and flux measures for event (**a**) CC than event (**b**) TS.

The (*t*, *f*) variance feature amongst the top ranked features in classifying the events of interest can be justified by the fact that the CC sound is an impulsive sound with a limited time duration, whereas the TS is a sustained sound that lasts for several seconds. The variance in the CC sound appears to be higher than the TS sound, due to the high nonuniformity in it. Similarly, the same justification is valid for the coefficient of variation in the (*t*, *f*) plane, which is the ratio of variance over mean, and appears in the second position in the list. The spectral skewness and spectral flatness measure the spread and uniformity of signal energy distribution in the joint (*t*, *f*) plane. Based on the energy distribution of signals in the (*t*, *f*) plane, they can be discriminated. For example, as illustrated in Figure 5, the energy of the TS sound in the (*t*, *f*) domain are concentrated along the IFs of the signal components, which results in lower values of the (*t*, *f*) spectral flatness, whereas the energy of the CC signal is more widely distributed in the (*t*, *f*) domain, resulting in higher values of (*t*, *f*) spectral flatness. Similarly, the extension of spectral entropy to (*t*, *f*) spectral entropy measures the randomness in the signal’s energy distribution in the (*t*, *f*) plane. The energy of the CC signals is more uniformly distributed in the (*t*, *f*) domain as compared to the TS signals, resulting in high TF entropy.

**Figure 6:**
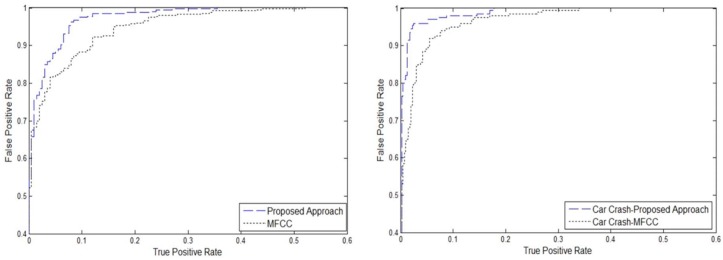
Receiver operating characteristic (ROC) curves of the proposed system configured with Mel-frequency cepstral coefficient (MFCC) features.

We can further compare the experimental results of our approach with that achieved using the MFCC features [47], by considering the receiver operating characteristic (ROC) curves depicted in Figure 6, which reflect the overall performance of the classification systems. The proposed set of features clearly outperforms the MFCC features, as the corresponding curve lies closer to the left and top borders of the quadrant. We can also consider the area under the ROC curve (AUC) as a measure of performance: the AUC results are reported in Table 5. The closer a ROC curve is to the top-left corner of the plane, the better is the performance of the corresponding classification system. Similarly, a higher AUC value indicates better overall performance, with a value of 1 indicating perfect classification. The proposed method (dashed line) outperforms the MFCC features (dotted line), with an AUC that is approximately 2% higher regardless of whether CC and TS are both considered as positive classes or CC alone is considered as the positive class. 

The proposed method detects audio events using an off-line signal analysis. However, in the future we aim to optimize the method for real-time audio signal event identification and classification. The TFDs provide a huge amount of information, which makes them computationally complex and less attractive for real-time application. However, the computational load of TFDs can be significantly reduced by using more computationally efficient algorithms for implementing TFDs [34]. The proposed approach takes 6 s in processing the three events, i.e., CC, TS and BN of duration 0.75 s each, using a PC equipped with an Intel Core i7, 64 GB of RAM with NVIDIA graphics. The computational time of training a classifier and feature selection is not included. The GPU systems can further reduce the computational time of our algorithm.

## 4. Conclusions

The ability to detect hazardous events on the road can be a matter of life and death, or it can mean the difference between a normal life and a life with a major handicap. In this work, we have proposed a system that combines *t*-, *f*- and (*t*, *f*)-domain features to simultaneously consider the non-stationary, instantaneous, and long-term properties of audio signals to facilitate automatic detection and classification of audio anomalies. The results of experiments performed on a publicly available benchmark dataset demonstrate the robustness of the proposed method against background noise compared with state-of-the-art approaches for road surveillance applications. Our audio classification system is confirmed to be effective in detecting hazardous events on roads, with a 7% improvement in accuracy rate compared with the state-of-the-art approaches.

## Figures and Tables

**Table 1:** Temporal and Spectral features.

Temporal Features	Spectral Features
The standard Mean of a time domain audio signal x[n] is given by,	The Spectral Flux can be estimated by taking the sum of absolute difference of the Fourier transforms (FTs) of the signal,
${\text{µ}}^{\left(t\right)}=\frac{1}{N}\sum_{n}x\left[n\right]$	$SF_{\left(f\right)}=\sum_{n=1}^{K/2}|Y_{x}\left[n\right]-Y_{x-1}\left[n\right]|$
The Variance of a time-domain is given as,	here $Y_{x}$ is the FT of the xth frame of the time-domain signal.
$\sigma^{2\left(t\right)}=\frac{1}{N}\sum_{n}{\left({\text{µ}}^{\left(t\right)}-x\left[n\right]\right)}^{2}$	The Spectral Entropy can be expressed as,
The Skewness measure of time-domain is represented as,	$SE_{\left(f\right)}=-\sum_{n=1}^{K}S_{x}\left[n\right]\;log_{2}(\left[S_{x}\left[n\right]\right]$
$\gamma^{\left(t\right)}=\frac{1}{N\sigma^{\left(t\right)3}}\sum_{n}{\left(x\left[n\right]-{\text{µ}}^{\left(t\right)}\right)}^{3}$	where $S_{x}\left[n\right]=\frac{\left|Y_{x}{\left[n\right]}^{2}\right|}{\sum_{n=1}^{K}|Y_{x}\left[n\right]{|}^{2}}$
The kurtosis measure of a time domain signal is given as,	The spectral Flatness is defined as the geometric means of the FT of a signal normalized by its arithmetic mean
$k^{\left(t\right)}=\frac{1}{N\sigma^{\left(t\right)4}}\sum_{n}{\left(x\left[n\right]-{\text{µ}}^{\left(t\right)}\right)}^{4}$	$SFT_{\left(f\right)}=K\;\frac{{\left(\Pi_{n=1}^{K}|Y_{x}\left[n\right]|\right)}^{K-1}}{\sum_{n=1}^{K}|Y_{x}\left[n\right]|}$
The coefficient of variation of a time-domain signal is given as,	here $Y_{x}\left[n\right]$ is the FT of the analytic associate y[n] of a real signal, and K is the length of signal $Y_{x}\left[n\right]$.
$C^{\left(t\right)}=\frac{\sqrt{\sigma^{2\left(t\right)}}}{{\text{µ}}^{\left(t\right)}}$	The Spectral Roll−off can be expressed as,
Energy Entropy of a time-domain signal ca be represented as,	$SR_{\left(f\right)}=\lambda \;\sum_{n=1}^{K}|Y_{x}\left[n\right]|$
$E^{\left(t\right)}=-\sum_{n}x\left[n\right]\;log_{2}\left(x\left[n\right]\right)$	where λ represents a threshold.
The Zero Crossing Rate of a time-domain signal can be represented as,	Spectral Centroid can be represented as,
$Z^{\left(t\right)}=\sum_{n}\left|sign\;\left[x\left[n\right]\right]-sign\;\left[x\left[n\right]\right]\right|w\left(m-n\right)$	$SC_{\left(f\right)}=\frac{\sum_{n=1}^{K}\;n|Y_{x}\left[n\right]|}{\sum_{n=1}^{K}|Y_{x}\left[n\right]|}$
where $w\left(m\right)=\left\{\begin{array}{cc} \frac{1}{2N} & 0\geq m\leq N-1\\ 0 & other\;wise \end{array}\right\},$ and sign represents is a sign function	Maximum power of the frequency bands can be represented as,
	$MP_{\left(f\right)}=\sum_{n}^{K}{\left|Y_{x}\left[n\right]\right|}^{2}$

**Table 2:** Details on the composition of the dataset.

Class	# Events	Duration (s)
CC	200	326.3
TS	200	522.5
BN	-	2737

**Table 3:** Ranking of the *t*-, *f*- and (*t*, *f*)-domain features based on mutual information criteria.

Time and Frequency Features	Time-Frequency Features	Selected Features
Mean (*t*)	Mean (*t*, *f*)	Variance (*t*, *f*)
Variance (*t*)	Variance (*t*, *f*)	Coefficient of Variation (*t*, *f*)
Skewness (*t*)	Coefficient of Variation (*t*, *f*)	Skewness (*t*, *f*)
Coefficient of Variation (*t*)	Skewness (*t*, *f*)	Flatness (*t*, *f*)
Kurtosis (*t*)	Kurtosis (*t*, *f*)	Spectral Entropy (*t*, *f*)
Energy Entropy (*t*)	Flatness (*t*, *f*)	Spectral Flux (*t*, *f*)
Zero Crossing Rate (*t*)	Renyi Entropy (*t*, *f*)	Instantaneous Amplitude (*t*, *f*)
Short-Time Energy (*t*)	Spectral Flux (*t*, *f*)	SVD-based Feature (*t*, *f*)
Flatness (*f*)	Instantaneous Frequency (*t*, *f*)	TFD Concentration Measure (*t*, *f*)
Spectral Entropy (*f*)	Instantaneous Amplitude (*t*, *f*)	Aspect Ratio (*t*, *f*)
Spectral Roll-off (*f*)	SVD-based Features (*t*, *f*)	Flatness (*f*)
Spectral Centroid (*f*)	TFD Concentration Measure (*t*, *f*)	Spectral Entropy (*f*)
Spectral Flux (*f*)	Area or Convex Hull (*t*, *f*)	Spectral Flux (*f*)
Maximum power	Aspect Ratio (*t*, *f*)	Spectral Centroid (*f*)
of the frequency bands (*f*)		Spectral Roll-off (*f*)
		Skewness (*t*)
		Kurtosis (*t*)
		Energy Entropy (*t*)
		Short-Time Energy (*t*)

**Table 4:** Classification matrices achieved using (**a**) the proposed approach, (**b**) the MFCC features proposed in [47], (**c**) the method defined in [13] with temporal and spectral features and (**d**) the method defined in [13] with MFCC Features using Bag of Word (BoW) approach.

		(a) Proposed Approach			(b) MFCC Features [47]		
		Predicted Class					
		TS	CC	BN	TS	CC	BN
True Class	TS	94%	3%	3%	85.5%	5%	9%
	CC	1.5%	96%	2.5%	0%	92.5%	7.5%
	BN	8%	2%	90%	5.5%	8.5%	86%
		**(c) Temporal and Spectral Features [13]**			**(d) MFCC Features (BoW) [13]**		
		**Predicted Class**					
		**TS**	**CC**	**BN**	**TS**	**CC**	**BN**
	TS	75.0%	0.5%	24.5%	71%	0.5%	28.5%
	CC	0%	89%	11%	1%	89.5%	9.5%

**Table 5:** Comparison of performance results between the proposed approach and other approaches.

Methods	RR (%)	MDR (%)	FPR (%)	AUC (%)
Bark Features [13]	78.20	21	10.96	86
MFCC Features_BoW [13]	82.65	19	5.48	90
Temporal and Spectral	84.5	17.75	2.85	80
Features [13]				
MFCC Features [47]	88	8.25	7	96.72
Proposed Approach	95	2.75	5	98.32

**Table 6:** Comparison of recognition rate achieved between the three typologies of features.

Features	RR (%)
Temporal	61
Spectral	81.5
Time-Frequency	84
Joint set of Features	93
Proposed set of features	95
